# Bilateral simultaneous thalamic hematomas – Unusual presentation of intracerebral hemorrhage: A case report

**DOI:** 10.1016/j.heliyon.2023.e20622

**Published:** 2023-10-04

**Authors:** Rita Magyar-Stang, Marcell László Palotai, Gertrúd Tamás, Judit Kárpáti, Péter Barsi, Dániel Bereczki, Bence Barna Gunda

**Affiliations:** a Semmelweis University, Department of Neurology, Budapest, Hungary; b Semmelweis University, János Szentágothai Doctoral School of Neurosciences, Budapest, Hungary; c Semmelweis University, Medical Imaging Centre, Department of Neuroradiology, Budapest, Hungary; d HUN-REN-SU Neuroepidemiological Research Group

**Keywords:** Hypertension, Intracerebral hemorrhage, Cognition disorder, Cerebral microangiopathy, Risk factors

## Abstract

**Background:**

Bilateral symmetrical simultaneous thalamic hemorrhages are extremely rare.

**Case presentation:**

A 52-year-old female patient with a history of untreated hypertension, ischemic heart disease and type 2 diabetes mellitus was admitted with somnolence, disorientation, 3/5 right-sided hemiparesis and blood pressure of 200/110 mmHg. Cranial CT scan showed bilateral thalamic hemorrhages, with bilateral intraventricular propagation and subarachnoid component along the frontal, parietal and occipital lobes. CT angiography did not show any source of bleeding or cerebral vein or sinus thrombosis. Coagulation laboratory parameters were in normal range.

The patient was treated with a combination of intravenous and oral antihypertensive medication; five days later she become normotensive with improving motor function but was still somnolent.

Six weeks later she was fully alert, motor functions continued to improve, but had severe cognitive deficit. Repeated neuropsychological assessment showed a slow and moderate improvement of a major neurocognitive impairment. At discharge her Mini Mental State Examination score was 13/30 and Addenbrooke's Cognitive Examination III score was 42/100.

Cranial MRI scan eight weeks later depicted subacute-chronic stages of the bilateral hemorrhages, regression of perifocal edema, cerebral microbleeds in the left external capsule and the pons.

At discharge after 2 months, she was alert, had no focal neurological signs, but was unable to care for herself due to lack of motivation, spatial and temporal disorientation and severe cognitive deficit.

**Conclusion:**

Simultaneous bilateral thalamic hemorrhages are extremely rare, the most commonly observed symptom is cognitive impairment. Our case was caused by hypertensive crisis, but in the differential diagnosis, sinus thrombosis, hemorrhagic transformation of ischemic stroke and various hemophilias should be considered.

## 1 Background

Intracerebral hemorrhage (ICH) has the highest mortality of all stroke subtypes reaching more than 50% at 1 year [1]. Basal ganglia, thalamus and brain stem are the typical localization of solitary hypertensive ICHs. However, bilateral symmetrical simultaneous thalamic hemorrhages are extremely rare and their pathophysiological background is not clear [2,3]. We present a case with bilateral thalamic hemorrhages, consider its possible mechanisms and provide a review of the literature.

### 1.1 Case presentation

A 52-year-old female patient with history of ischemic heart disease and untreated essential hypertension for decades was admitted with somnolence, disorientation, 4/5 right-sided hemiparesis and high blood pressure (200/110 mm Hg).

Based on reports from her relatives, her family history was only positive for essential hypertension (no other cardio- or cerebrovascular diseases) and at the time of admission the non-compliant patient had not been taking any of her prescribed medications for a long time (including antihypertensives or antithrombotics).

**Fig. 1:**
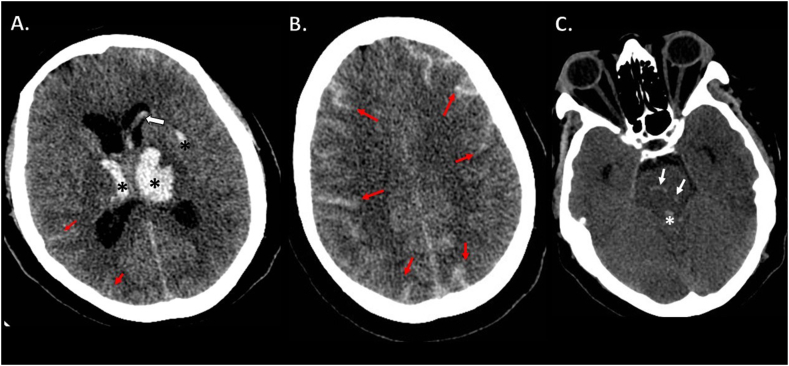
CT scans of bilateral thalamic hemorrhages. A: axial CT scan at thalamic level depicts bilateral thalamic and left external capsule hemorrhages (black stars), intraventricular propagation (white arrow) and subarachnoid hemorrhage (red arrows). B: axial CT scan at centrum semiovale level shows extensive subarachnoid hemorrhage components (red arrows). C: axial CT scan depicts small hemorrhages in the mid-pons region (white arrows) and hemorrhage in the 4th ventricle (white star).

Urgent non-contrast cranial computer tomography (CT) scan showed bilateral thalamic hemorrhages, accompanied by bilateral intraventricular propagation [Fig. 1 (A)] and subarachnoid component along the frontal, parietal and occipital lobes [Fig. 1 (B)]. Small pontine hemorrhages were also seen [Fig. 1 (C)]. CT angiography did not show any active contrast extravasation or macrovascular source of bleeding. There were no signs of sinus or deep cerebral vein thrombosis. Coagulation laboratory parameters (international normalized ratio (INR), activated partial thrombin time (APTT), prothrombin time (PT), platelet count) were in normal range. On ECG there was normofrequent sinus rhythm. HbA1c was 5,7%.

The patient was admitted to our neuro-intensive care unit (NICU) and intravenous urapidil (50 mg/50 ml with a dosing rate of 5 ml/h in a perfusor) was initiated within 6 hours of the symptom onset, with the target goal of systolic blood pressure reduction between 130 and 150 mm Hg. On the next day additional combination oral antihypertensive medications (1 × 20 mg enalapril, 2 × 5 mg amlodipine, 1 × 2 mg doxazosin) were initiated. On the following days the dose of iv. urapidil was gradually reduced, and on the 5th day of administration it could be stopped. Hypertensive crisis did not occur again during her hospital stay.

On the 5th day the patient was transferred from NICU to our regular Stroke Unit in a normotensive, stable condition, her hemiparesis improved to 3/5 and was still somnolent.

Six weeks after admission she became fully alert, right-sided hemiparesis gradually improved.

Follow-up CT scans on day 4, 13, 25 showed a slow, but steady regression of the hemorrhages.

Due to her mental status and decreased level of consciousness, she was initially unsuitable for a detailed cognitive evaluation. Later repeated neuropsychological assessment at week 1, 2, 4 and 6 showed a slow and moderate improvement of a major neurocognitive impairment. During this time, she received cognitive rehabilitation by a clinical psychologist. At discharge her Mini Mental State Examination score was 13/30 and Addenbrooke's Cognitive Examination II score was 42/100.

**Fig. 2:**
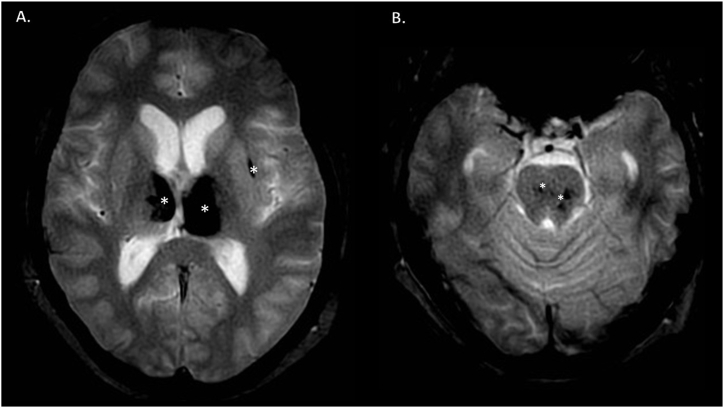
MRI T2* Fast field echo (FFE) of bilateral thalamic and pons bleeding. A: bilateral thalamic and external capsule bleeding (white stars). B: small hemorrhages in pons (white stars).

Cranial magnetic resonance imaging (MRI) scan on the eighth week depicted subacute-chronic stages of the bilateral thalamic hemorrhages, regression of perifocal vasogenic edema, 1 cerebral microbleed in the left external capsule and 3 in the pons. Leukoencephalopathy or any other underlying abnormality was not seen. [Fig. 2 (A, B)]

At discharge she was in normotensive, alert, had no focal neurological signs, walked on her own without any aid but was unable to care for herself due to lack of motivation, spatial and temporal disorientation and cognitive deficit. The patient was eventually discharged after 2 months to a chronic care unit and placed under guardianship as she remained incapacitated.

At discharge combined antihypertensive medication ACE-inhibitor (enalapril), calcium channel blocker (amlodipine), alpha-adrenergic receptor blocker (doxazosin) and diuretic (indapamide) were prescribed for secondary prevention. In her discharge documentation we highlighted the importance of the strict blood pressure control (target blood pressure: <140/90 mm Hg, daily blood pressure measurement, keeping documented blood pressure diary) for her family doctor and caregiver staff in order to avoid another possible hypertensive crisis.

## 2 Discussion

The incidence of multiple ICH is between 0.9 and 4.7% of all ICHs on a global scale [3].

Multiple simultaneous hemorrhages in extrathalamic regions usually affect the basal ganglia, lenticulostriatal region, putamen, caudate nucleus, internal and external capsule with characteristic neurological symptoms [4]. Thalamoperforating arteries are responsible for thalamic hemorrhage while lenticular nuclei hemorrhages originate from lenticulostriate arteries from the anterior circulation [5].

Bilateral, simultaneous bleeding, affecting only the two thalami are extremely rare. Choudhary et al. summarized 9 cases of simultaneous bilateral hemorrhages of the deep hemispheric structures [2]. Perez et al. reported not simultaneous but subsequent cases of bilateral thalamic hemorrhages [6], previously Imai et al. collected radiographic features of 10 simultaneous thalamic hemorrhage cases [7]. We summarized in Table 1 simultaneous bilateral thalamic hemorrhage cases published so far with their etiological and outcome characteristics. Together with our case, 25 simultaneous bilateral thalamic hemorrhage cases have been reported so far.

**Table 1:** Summary of bilateral thalamic hemorrhage cases.

Authors, year	Cause of hemorrhage	Outcome
Tanikake et al., 1983 [8]	Hypertension	Coma
Hickey et al., 1988 [9]	Hypertension	Death at 1 month
Yabzmoto et al. [10], 1989	Acute myeloblastic leukemia	Death in 1 week
Tanno et al., 1989 [11]	Hypertension	severe consciousness disturbance
Erbguth et al., 1991 [12] (3 cases)	Cerebral deep vein thrombosis	2 cases: slight aphasia and hemiparesis 1 case: slight coreatic movement disorder of the right hand
Lin et al., 1993 [13]	Hypertension	Death at 1 week
Kabuto et al., 1995 [14]	Hypertension	Vegetative state
Wang et al., 1995 [15]	Cerebral deep vein thrombosis	confusion and adversive seizures
Dromerick et al., 1997 [16]	iv. rtPA administration	paramedian diencephalic syndrome
Sunada et al., 1999 [17]	Hypertension	Death at 6 months
Imai K., 2000 [7] (4 cases) [7]	Hypertension	Apallic syndrome, skew deviation, left hemiparesis
Yen et al., 2005 [18] (2 cases)	N/R	activity of daily life according to Kanaya's grading system 4-5
Choi et al., 2005 [19]	Hypertension	Drowsy mental state
Takeuchi et al., 2011 (2 cases) [20]	Hypertension	Death
Kono et al., 2013 [3]	Hypertension	mild right hemiparesis mRS 1 et 3 months
Seo et al., 2014 [5]	N/R	Death
Choudhary et al., 2018 [2]	Hypertension	right hemiplegia, mRS 4 et 2 weeks
Present case	Hypertension	lack of motivation, spatial and temporal disorientation and severe cognitive deficit

N/R = not reported; mRS = modified Rankin scale.

### 2.1 Causes of ICH

Most commonly (80%), rupture of cerebral small arterioles causes intraparenchymal bleeding. Deep perforator arteriopathy (arteriolosclerosis or hypertensive arteriopathy) leads to vulnerable microaneurysms and a subsequent rupture of the vessel wall. Deep perforator arteriopathy is linked to hypertension and is a common cause of basal ganglia and brainstem ICH, but also contributes to lobar hemorrhages [3].

**Fig. 3:**
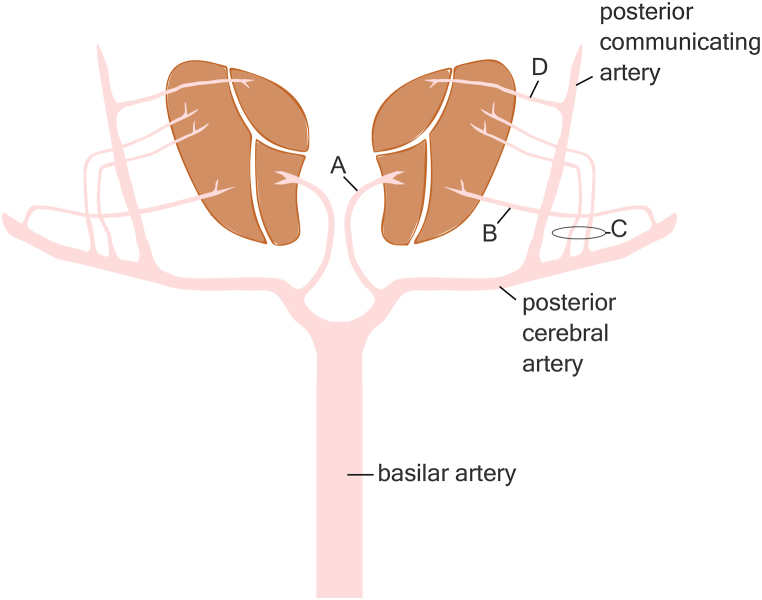
Arterial blood supply of the thalami. A: paramedian artery; B: posterior choroidal artery; C: thalamogeniculate arteries; D: tuberothalamic artery.

Fig. 3 depicts the arterial blood supply of the thalami. Thalamic hemorrhages of hypertensive origin probably originate from these deep perforating branches (Fig. 3.).

Leptomeningeal perforator arteriopathy is characteristic for cerebral amyloid angiopathy mostly resulting in lobar intracerebral hemorrhages and convexity subarachnoid hemorrhages. A small proportion (20%) of ICHs are caused by macrovascular bleeding sources such as cavernomas, arteriovenous malformations or fistulas.

In Table 1 simultaneous bilateral thalamic hemorrhages etiological factors are summarized. The most common etiological factor was hypertension (72%), in 1 case leukemia (4%), in 2 cases (8%) deep cerebral vein thrombosis and in 1 case (4%) a complication of iv. thrombolysis caused bilateral thalamic bleeding. In 3 cases (12%), the underlying cause could not be clearly identified.

### 2.2 Possible mechanisms of bilateral hemorrhages

There are a few possible explanations for bilateral hypertensive hemorrhages. The first hypothesis concerns the rupture of vulnerable perforator vessels, which is followed by a systemic increase in blood pressure, that subsequently causes bleeding on the opposite side. This hypothesis is supported by the observation that in some cases motor symptoms initially develop unilaterally before the onset of tetraparesis.

Our previous case report of a hypertensive putaminal ICH serendipitously captured by serial CT along with other observations suggests that even in a solitary ICH there are multiple sources of bleeding due to a cascade of secondary vessel ruptures induced by a tissue shockwave with excentric expansion [[21], [22], [23], [24], [25]]. This mechanism may cause an initially unilateral hemorrhage to spread to adjacent brain areas and become bilateral.

According to another explanation, the perforator vessels are generally vulnerable and in rare cases rupture occurs on both sides coincidentally possibly due to a surge in blood pressure [2].

Bilateral simultaneous thalamic ischemia is caused by Percheron artery occlusion (bilateral thalamic perforators from a single stem), or bilateral embolism to straight thalamic perforators from the bifurcating basilar artery; its hemorrhagic transformation is extremely rare [26]. In our case the massive hematoma from onset and rupture into the ventricles make this etiology very unlikely, however MRI was performed relatively late, so an ischemic origin cannot be excluded completely.

Hemorrhagic transformation of cerebral venous infarcts are common in superior sagittal sinus and cortical vein thrombosis. The internal veins draining the thalami are affected in only 6% of cerebral venous thrombosis. (12) Only two case presentations report secondary bilateral thalamic hemorrhagic transformation in deep vein and sinus thrombosis. (7) In the case of deep venous thrombosis, bleeding is caused by an increase in venous pressure, which first causes vasogenic edema. Later cytotoxic edema and venous infarction develop, and finally the process leads to hemorrhagic transformation. The topography of the damage is determined by the supply areas of the draining deep cerebral veins (great cerebral vein of Galen, internal cerebral veins, thalamostriate veins and choroidal veins). These areas are the thalami and basal ganglia. [27].

Our patient most likely suffered a spontaneous hypertensive multifocal intracerebral hemorrhage with extension into the ventricular system and the subarachnoid space. We consider the small pontine hemorrhages as secondary to a sudden increase in intracranial pressure due to the supratentorial hemorrhage (Duret bleeding), since pontine bleedings were already identifiable on the CT scan in the acute stage. Duret hemorrhage occurs when the brainstem is displaced rapidly inferiorly due to trauma, increased intracranial pressure or surgery. At the same time perforating vessels of the basilar artery and paramedian pons remain relatively fixed; this may cause the perforating arteries to shear and lead to hemorrhage [28].

In our case the possibility of a further systemic acute hypertensive response to the intracerebral hemorrhage can be assumed. Factors typically associated with acute hypertensive response [29] that could also be identified in the present case are: untreated chronic hypertension, increased intracranial pressure, brainstem bleeding (causing possible autonomic dysregulation), temporary iv. use of antihypertensive medication for a few days.

### 2.3 Clinical characteristics of bilateral thalamic lesions

Various degrees of hypnoid disturbances of consciousness (from coma to somnolence) can occur in the acute phase of a bilateral thalamic insult due to damage to the ascending reticular activation system (ARAS) [30]. Thalamus dementia (paramedian diencephalon syndrome) becomes apparent in later phase due to disturbances of limbic and frontal connections. These patients have inattention, visuoperceptual disturbance, executive dysfunction, memory disturbances, anterograde or/and retrograde amnesia. Dromerick et al. reported paramedian diencephalon syndrome after bilateral thalamic hemorrhage [16]. Our patient showed a similar clinical course with an initial decreased conscious level and subsequent thalamus dementia.

Irregular alpha activity (alpha coma) and external stimuli elicited theta-delta waves (paradoxical activation) are characteristic findings on electroencephalogram (EEG) caused by bilateral thalamic lesions [9]. Unfortunately, in our case the patient's thick hair did not allow the placement of the electrodes, and after gradual improvement in her level of consciousness, we did not force EEG examination that would have required the shaving of the lady's hair.

The clinical characteristic of bilateral lenticular nuclei bleeding is somewhat different, in these cases acutely altered mental status, hemi- or quadriparesis, and at a later stage symptoms of parkinsonism can be detected [4,5].

## 3 Conclusion

Simultaneous bilateral thalamic hematomas are extremely rare. The most common cause is hypertensive small vessel disease, however mechanisms for bilateral presentation are still unclear. The most important differential diagnoses are deep cerebral venous thrombosis with bilateral venous hemorrhagic infarct due to increased venous pressure and edema in the thalamic and basal ganglia regions, and bilateral ischemic stroke with hemorrhagic transformation. The clinical presentation is dominated by impaired consciousness in the acute and major cognitive disturbances in the later phase for which cognitive rehabilitation has a paramount importance for these patients.

## Author contribution statement

All authors listed have significantly contributed to the investigation, development and writing of this article.

## Data availability statement

Data will be made available on request.

## Declarations

Ethics approval and consent to participate: We confirm that we have read the journal's position on issues involved in ethical publication and affirm that this report is consistent with those guidelines. As this is a case report describing clinical observations, ethics approval was waived.

Consent for publication: The patient gave written consent for their personal or clinical details along with any identifying images to be published in this study.

Availability of data and materials: The materials used and presented during the present case report are available from the corresponding author on reasonable request.

## Funding

None.

## Declaration of competing interest

The authors declare that they have no known competing financial interests or personal relationships that could have appeared to influence the work reported in this paper.

## Abbreviations

ARAS: ascending reticular activation system
CT: computer tomography
EEG: electroencephalography
FFE: Fast field echo
ICH: intracerebral hemorrhage
MRI: magnetic resonance imaging
mRS: modified Rankin scale
NICU: neuro-intensive care unit
rtPA: recombinant tissue plasminogen activator
